# Phellinus Linteus Extract Sensitizes Advanced Prostate Cancer Cells to Apoptosis in Athymic Nude Mice

**DOI:** 10.1371/journal.pone.0009885

**Published:** 2010-03-31

**Authors:** Takanori Tsuji, Wei Du, Takashi Nishioka, Lihua Chen, Daisuke Yamamoto, Chang Yan Chen

**Affiliations:** Department of Radiation Oncology, Beth Israel Deaconess Medical Center, Harvard Medical School, Boston, Massachusetts, United States of America; Health Canada, Canada

## Abstract

Phellinus linteus (PL) mushroom possesses anti-tumor property. We previously reported that the treatment with PL caused cultured human prostate cancer cells to undergo apoptosis. To further studying the mechanisms of PL-mediated apoptosis, we performed xenograft assay, together with *in vitro* assays, to evaluate the effect of PL on the genesis and progression of the tumors formed from the inoculation of prostate cancer PC3 or DU145 cells. After the inoculation, nude mice were injected with PL every two days for 12 days. Although PL treatment did not prevent the formation of the inoculated tumors, the growth rate of the tumors after PL treatment was dramatically attenuated. We then tested the effect of PL on the tumors 12 days after the inoculation. After inoculated tumors reached a certain size, PL was administrated to the mice by subcutaneous injection. The histochemistry or immunochemistry analysis showed that apoptosis occurred with the activation of caspase 3 in the tumors formed by inoculating prostate cancer DU145 or PC3 cells. The data was in a good agreement with that from cultured cells. Thus, our in vivo study suggests that PL not only is able to attenuate tumor growth, but also to cause tumor regression by inducing apoptosis.

## Introduction

Prostate cancer is a devastated disease in men. Although therapies (such as surgery or androgen ablation) have improved the survival rate of the patients, lack of success with hormone-refractory prostate cancer remains. The advanced tumors are often resistant to chemotherapeutic drugs. Although the mechanisms for the resistance are unclear, it has been postulated that the development of tumors, such as refractory prostate cancer, is related to the accumulation of genetic or epigenetic alterations. In Asian, PL is one of well-established medicinal mushrooms that have been using to treat various human malignancies. Studies showed that the water-soluble fraction of PL is biologically active, which probably is polysaccharide [1]–[3]. It has also been shown that PL is able to suppress tumors *in vitro* either indirectly by enhancing the host's immune system, or directly by inducing apoptosis in tumor cells [4]–[6]. Recently, we reported that PL is remarkably effective in inhibiting the growth of various prostate cancer cell lines without toxic effects on normal prostate epithelial cells [7]. Since PL possesses the anti-tumor, anti-angiogenic and immunomodulatory properties, it has drawn wide interests in Asia to develop it for anti-cancer therapeutics. In order to develop this medicinal mushroom as anti-prostate cancer remedy, a better understanding of the underlying molecular mechanisms of PL functions is required.

A potential of a drug to induce apoptosis has been widely used as a strategy for developing new cancer therapy. It is known that apoptosis can be triggered by a variety of internal or external signals. Caspases, a highly conserved family of cysteine proteases, are key apoptotic effectors in cells and play critical roles in the regulation of apoptosis through a chain cleavage reaction [8],[9]. Caspases exist as pro-enzymes in the cytosol of cells and are activated through proteolysis. Upon cell death stimulations, caspase 8, as an initiator, triggers the activation of downstream effector caspases 3 and 7, resulting in BID cleavage and cytochrome c release [10]–[12]. The release of cytochrome c from the mitochondria to the cytosol causes the formation of the apoptosome, which in turn triggers the intrinsic apoptotic machinery. PL has been shown to cause prostate cancer cells to undergo apoptosis by triggering caspase cascade [6],[7]. Furthermore, low doses of doxorubicin (an anti-cancer drug), together with low concentrations of PL, have a synergistic effect on the induction of apoptosis in human prostate cancer cells [13].

Previously, we have reported that PL is able to sensitize cultured prostate cancer cells to apoptosis, in which caspases were activated [7]. Our present study, as the continuation of the investigation of the anti-tumor property of PL, further demonstrated the ability of PL to induce apoptosis in the tumors by inoculating prostate cancer DU145 or PC3 cells in nude mice. We showed that the injection of PL dramatically attenuated the genesis of the tumors in nude mice after the inoculation. Our study also demonstrated that upon PL injection, the formed tumors by inoculating DU145 or PC3 cells into nude mice underwent a regression, by triggering an apoptotic processes.

## Methods

### Cells and reagents

*Phellinus Linteus* powder was purchased from Panbio-Tech (Taejon, South Korea) and dissolved in water. After boiled for 5 min, PL solution was centrifuged and supernatant was collected for the study. DMEM (Dulbecco's modified Eagle's medium); antibiotics (penicillin and streptomycin) and trypsin-EDTA were purchased from Invitrogen. Human prostate cancer PC3 cells (American Type Culture Collection) were cultured in DMEM medium supplemented with 10% heat-inactivated fetal calf serum, 2 mM L-glutamine, 100 unites/ml of penicillin, 100 µg/ml of streptomycin.

### Xenograft Assay

Male nude mice (NCRNU-M-M, CrTac: Ncr-Foxn1nu) (Taconic) at ages of 4–6 weeks were used. Human prostate cancer PC3 cells (5×106) in 100 µl PBS were inoculated into the right flank area of each mouse. Three mice were served as a positive control. For the inhibition of tumor formation, 6 mice were treated with PL (30 mg/kg in 100 µl PBS) right after the inoculation and subsequently administrated the same amount of PL every 2 days. Another 3 mice were treated with equal amount of control Hitachi mushroom solution following the same time course as that used for PL. The sizes of the tumors were measured every 4 days with a caliper and calculated according to Tamayko's formula [14]. For monitoring tumor regression, after the implanted tumors reached about 1.0 cm in diameter, the mice were treated with either PL (30 mg/kg) or the same amount of the control mushroom solution every 2 days. Ten days later, the mice were sacrificed and the tumors were isolated for histopathology or histochemistry analysis.

### Histopathology or immunohistochemistry analyses

Isolated tumors were fixed in 4% phosphate-buffered neutral paraformaldehyde solution (pH 7.4). The histological specimens were sectioned and stained with hematoxylin/eosin (H&E), TUNEL or corresponding antibodies (Santa Crutz Biotec.).

### Statistic analysis

Means and standard deviations of the results of the experiments were computed. Standard deviations are displayed as error bars in the figures. A Student's T test was used and a *p* value of <0.05 was considered significant.

## Results

### PL attenuates DU145 or PC3 cells to form tumors in nude mice

**Figure 1:**
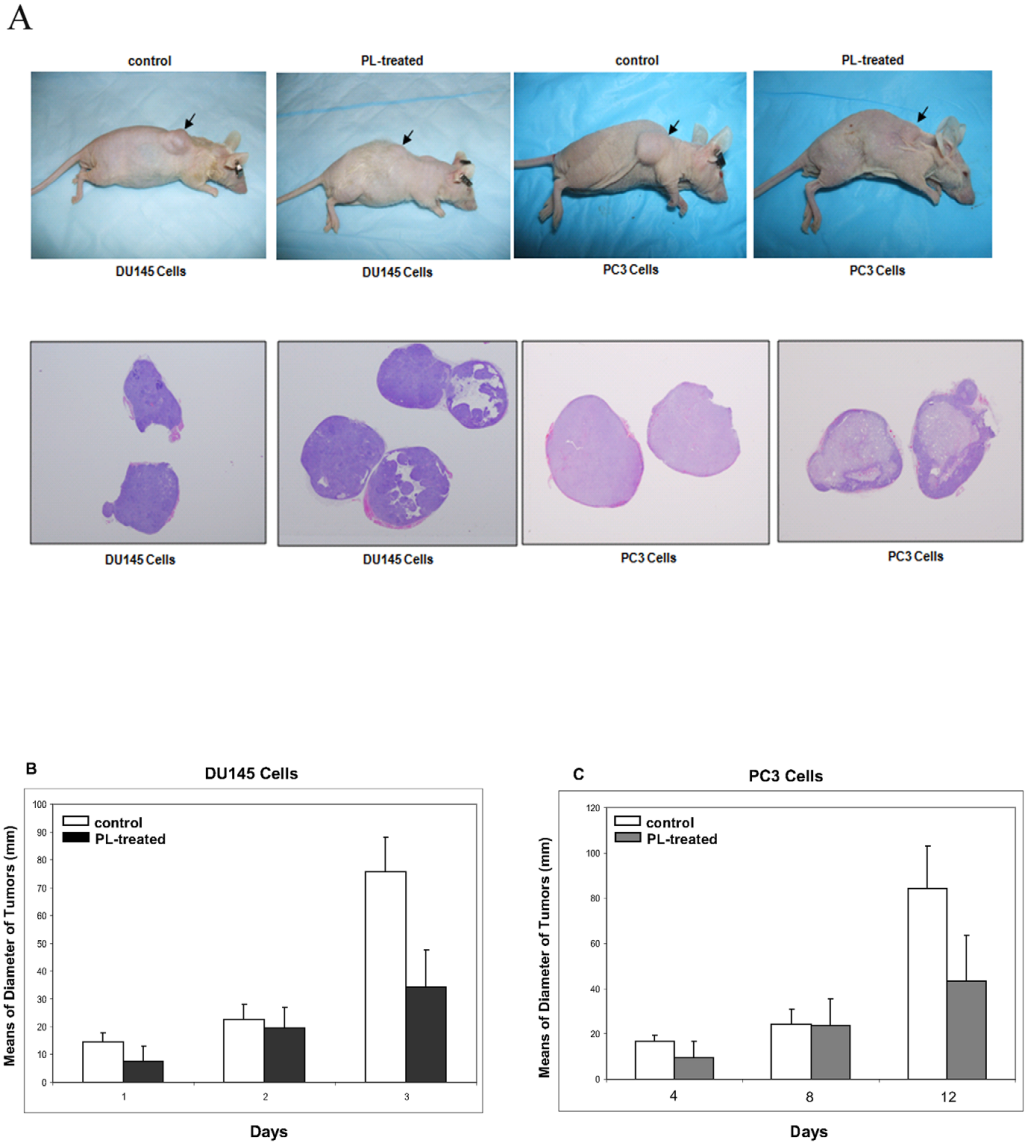
Attenuation of the tumors formed by the inoculation of DU145 or PC3 cells into nude mice after the treatment with PL. **A**. DU145 or PC3 cells were inoculated subcutaneously into nude mice. Twelve days later, the photos of the mice bearing the inoculated tumors were taken (upper panel). The tumors were isolated from PL treated or control mice. The slides mounted with the tumor tissues were prepared and stained with HE dye (lower panel). **B** and **C**. A group of 6 mice were injected with PL (30 mg/kg) in water from day 0 and subsequently administrated every 2 days. Another group of 3 was as the controls. One week later when the inoculated tumor started to form, the sizes of the tumors were measured every 4 days.

Studies have suggested that PL possess an anti-tumor property [1],[6],[7]. We demonstrated that PL not only is able to initiate apoptosis in various malignant prostate cancer cell lines, but induce G_1_ arrest in normal prostate epithelial cells [6],[7],[13]. To further test the anti-tumor effect of PL, xenograft assay was employed. After inoculated human prostate cancer PC3 cells into nude mice subcutaneously, PL was simultaneously injected into the mice. Afterwards, the same amount of PL was injected to the mice every two days. Twelve days later, the photos of the mice bearing tumors were taken (**Fig. 1A**, upper panel) and the slides mounted with tumor tissues were stained with HE (**Fig. 1B**, lower panel). The tumors in PL treated mice were much smaller than that in the control mice, and showed a distinct pattern of HE staining.

**Figure 2:**
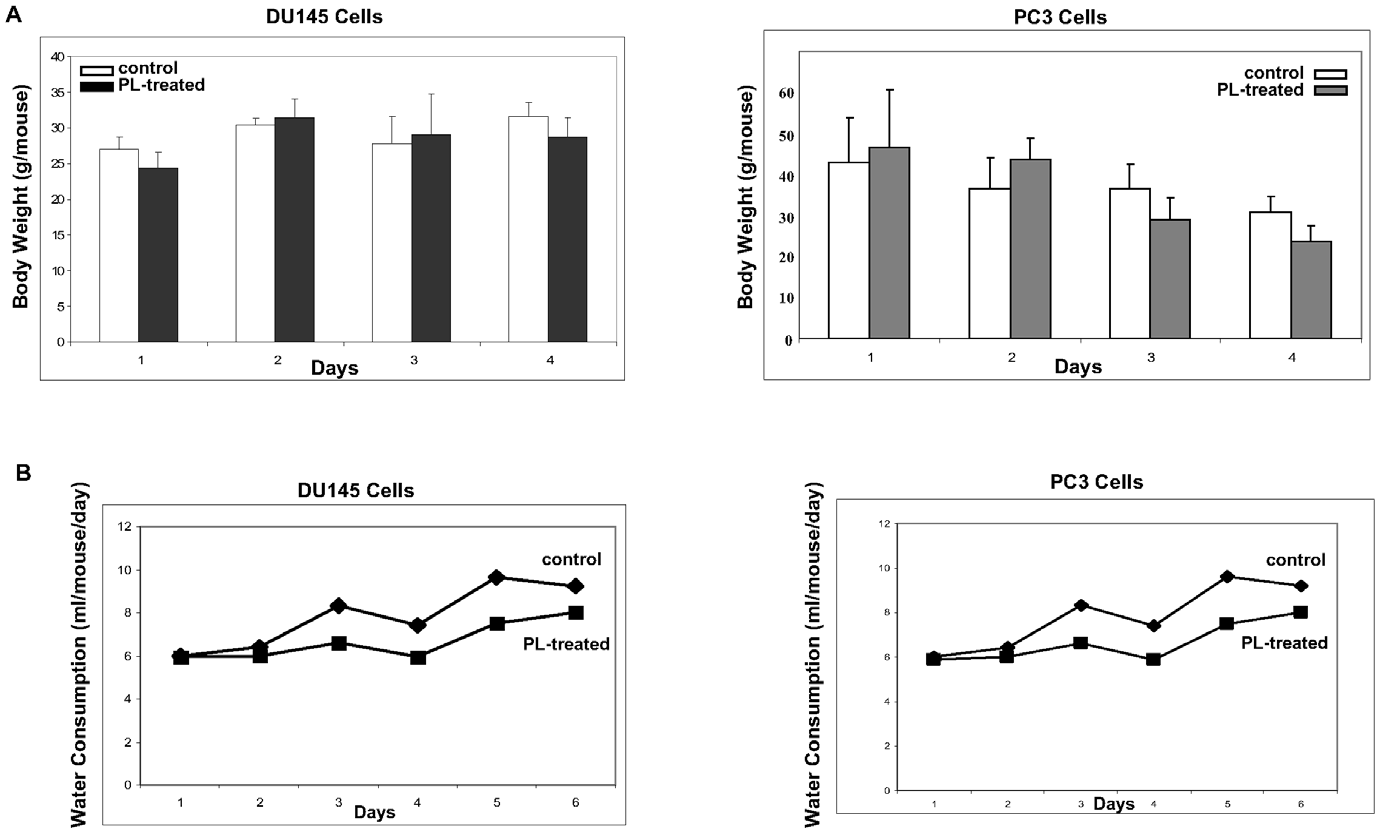
Analysis of side-effects of PL. **A** and **B**. After the tumor nodules were formed, the body weight (**A**) and water consumption (**B**) of each mouse with or without treated with PL were measured. Error bars represent the standard deviation from 3–6 mice.

We also measured the diameters of the inoculated tumors, one week later after the inoculation when all the mice started to show the sign of the formation of solid tumor masses. The measurements were taken every four days for 12 days (**Fig. 1B and C**). At day 12, the control tumor messes from the inoculation of two cell lines were about more than 2 folds bigger than those treated by PL. To exclude the possibility of toxicity rendered by PL, the weight (**Fig. 2A**) and water consumption (**Fig. 2B**) of the mice were measured and compared. The PL-treated mice had a similar weight or water consumption as the controls, suggesting that PL did not have a side-effect on treated or untreated mice. To further determine whether PL treatment had any toxic effects on the mice, the livers were also isolated out from the animals. The histochemistry staining with HE dye showed that the structures of liver from PL-treated mice were similar as that isolated from the control mice (data not shown). Both livers appeared healthy without any evidence of degeneration, fibrosis, apoptosis or necrosis. The in vivo study suggests that PL is able to attenuate the speed of the growth of the inoculated tumors in nude mice.

### PL treatment causes the growth regression in the tumors inoculated by prostate cancer cells

**Figure 3:**
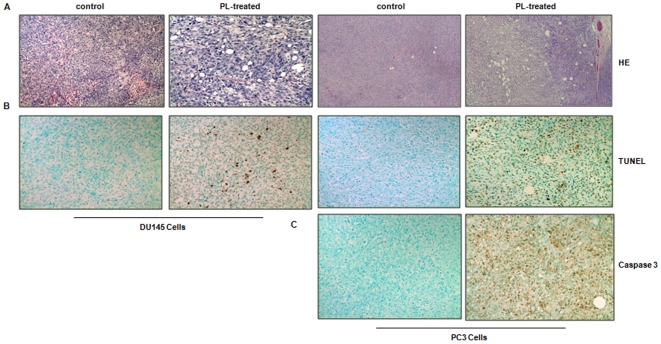
Histopathology or immunohistochemistry staining of PL-treated or control tumors isolated from the mice. **A**. The tumors were prepared for hematoxylin and eosin staining. **B** and **C**. The slides mounted with the tumor tissues were stained with TUNEL agent (**B**) or anti-caspase 3 antibody (**C**).

We then tested the effect of PL on the existing tumors. When the inoculated tumors reached to the size of 1.0 cm in diameter, PL was injected into the mice every two days for total ten days. The tumors were isolated and prepared for histopathological or immunohistochemitry analysis. The HE staining revealed that the tumors isolated from PL- or control mouse were well-circumscribed with connective tissues (**Fig. 3A**). Cell death appeared to occur in PL-treated tumors from the inoculation of two different prostate cancer cell lines with many unstained vacuoles, which was absent in the control tumors. To further determine whether apoptosis occurs in PL-treated tumors, immunohistochemistry analysis using TNUEL staining (to detect DNA strand breaks) was performed (**Fig. 3B**). The slides from PL-treated tumors were strongly stained with TUNEL staining, but only a few TUNEL staining positive cells were detected in control tumors. The percentages of the cells stained TUNEL positively was measured in the control and PL-treated tumors (data not shown). More than 30% of cells in PL-treated tumor had broken DNA strand and very small percent of cells from the control tumors (about 5%) stained positively. The immunohistochemistry staining using anti-caspase 3 antibody was also performed to detect the expression of caspase 3 (**Fig. 3C**). Large numbers of the cells in PL-treated tumor formed by the inoculation of PC3 cells were reacted with the antibody. In comparison, only a few cells in the control tumor expressed a high level of caspase 3. Similar results were obtained from the tumors formed by the inoculation of DU145 cells (data not shown). Thus, the results indicate that cell death occurred in PL-treated tumor is through apoptosis.

**Figure 4:**
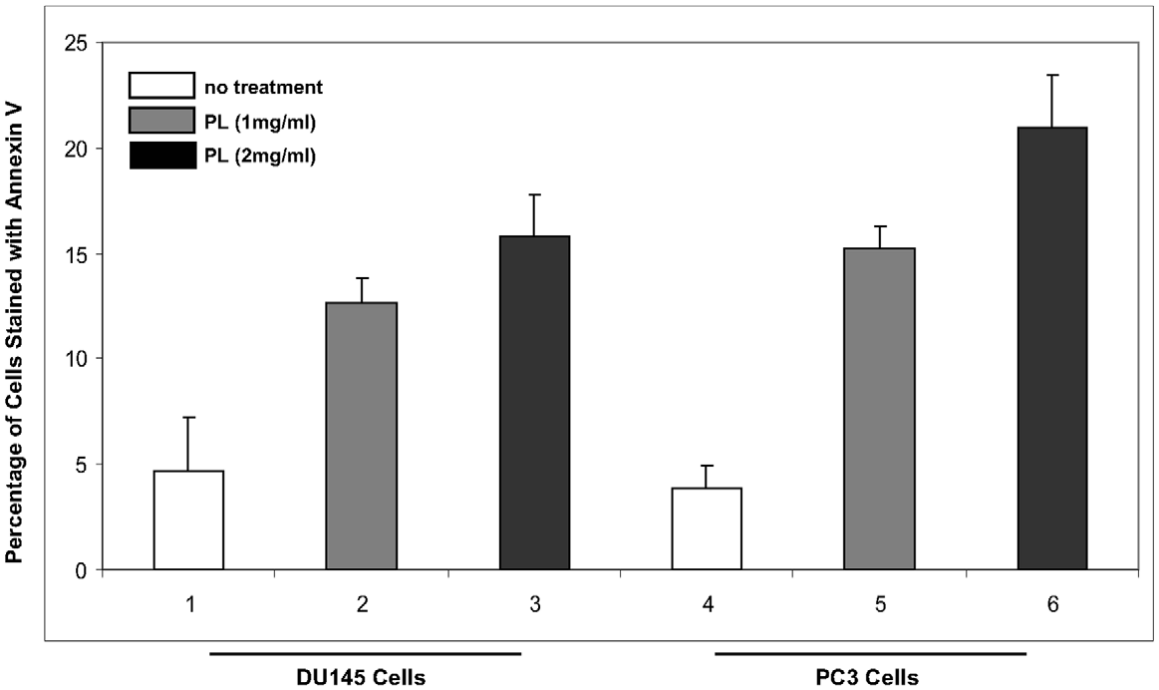
Induction of apoptosis in cultured DU145 and PC3 cells in response to PL treatment. The cells were treated with 1 mg/ml or 2 mg/ml for 48 h, and subsequently stained with Annexin V. Error bars represent the standard deviation from 3 independent experiments.

To further test the ability of PL to induce apoptosis, DU145 and PC3 cells were treated with PL at 1 mg/ml or 2 mg/ml for 48 h. Annexin V analysis were conducted (**Fig.** 4). After being treated with PL, about 15% of PL-treated cells underwent apoptosis, in a dose-dependent fashion. A baseline staining of Annexin V was seen in the control tumors. Interestingly, PC3 cells were more sensitive to PL than DU145 cells. Overall, the *in vitro* data is consistent with that obtained from the animal experiments.

## Discussion

Previously, we demonstrated that PL induces cultured prostate cancer cells to undergo apoptosis [7],[13]. Here, using xenograft assay, we showed that PL is capable of eliciting an apoptotic response *in vivo* to block the growth of the tumors formed by the inoculation of human prostate cancer DU145 or PC3 cells. The histochemistry staining analysis showed that the majority of the cells in the tumors isolated from PL-treated mice are apoptotic, manifested by positively stained with TUNEL and reacted with an anti-caspase 3 antibody. The occurrence of apoptosis was also demonstrated in cultured DU145 or PC3 cells after the treatment with PL for 48 in a dose dependent manner. Thus, our study, using *in vivo* as well *in vitro* assays, inidcates that PL treatment initiates caspase cascade to induce apoptosis in prostate cancer cells, which provide a strong evidence for the potential of PL as an anti-prostate cancer remedy.

It is known that PL is non-toxic in general [1]. Previously, we reported that PL, at high doses, induces control lung epithelial cells to arrest in the G_1_ phase of the cell cycle by blocking the expression of cyclin D_1_ and its interaction with cell cycle-dependent kinases 4 and 6 [6]. In our current study, the data from histopathological analysis demonstrate that the injection of PL into nude mice inoculated with either DU145 or PC3 prostate cancer cells has no toxic effect on the mice, manifested by that the treatment plays no role in the growth or water consumption of the mice. Importantly, the liver samples from the treated mice are normal without degeneration or fibrosis. It is possible that, like *in vitro* experiments, treatment with PL elicits cell checkpoint controls in normal tissues or organs, which is not harmful to the mice. In the inoculated tumors, the same treatment appears to be very much cytotoxic.

Programmed cell death is important machinery for excluding abnormal or cancerous cells. Activation of caspase family members is at the core of apoptosis, representing a point of intersection of various apoptotic pathways in various types of cancer cells, including prostate cancer cells. TNF or Fas/CD95 receptors, mitochondrial proteins (such as cytochrome c) and granzyme are able to induce cell death through activation of the caspase cascade [8],[9]. Damage to or stress in the ER or Golgi has been shown to be able to trigger apoptosis [15],[16]. Studies have shown that the unfolded protein response or lack of calcium is responsible for ER-mediated apoptosis [15]. ER stress has been demonstrated to cause the translocation of certain caspases to the ER or Golgi and the execution of apoptosis there. Calcium released from the ER during times of ATP deficiency is an important element in apoptosis induced by ischemia-reperfusion injury. We have shown that PL treatment triggers caspase activation in cultured prostate cancer cells [7],[13]. Our *in vivo* study indicates that PL treatment is also able to initiate apoptosis via activating caspase 3 that are the effector in caspase chain reaction. The investigation of whether other caspases are involved in the induction of apoptosis *in vivo* is under way.

PL is one of well-established medicinal mushrooms that have been taken orally as a common health-promoting dietary supplement or an adjuvant to treat malignancies in Asia for many decades. The extraction from water-soluble PL fraction shows a relatively homogeneous molecular weight distribution on gel permeation HPLC and is estimated to be around 150–180 kD from the retention time on HPLC pullulan molecular markers [1]. The main components of PL have been suggested to be various polysaccharides [1]–[3]. It has also been shown that the water soluble fraction of PL is able to suppress tumors either indirectly by enhancing the host's immune system, or directly by inducing apoptosis in tumor cells [4],[5]. However, it is still unclear which compositions of polysaccharide in PL possess the anti-cancer effect.

In summary, the successful use of PL to treat prostate cancer requires the fully understanding of the mechanisms for how PL mediates anti-tumor activities in tumors. Our previous study demonstrated that PL, through activation of caspases, is able to induce apoptosis in cultured cancer cells. In the present investigation, using xenograft assay, we further demonstrate that the injection of PL into nude mice with formed subcutaneous tumors by inoculated with prostate cancer DU145 or PC3 cells induces majority of inoculated tumor cells to lose their viabilities. Thus, our *in vivo* data support the notion that PL, by switching on a tumor suppression-related machinery, can be developed as an efficient therapy to treat human malignancies, such as refractory prostate cancer.

## References

[1] Song KS, Cho SM, Lee JH, Kim HM, Han SB (1995). B-lymphocyte-stimulating polysaccharide from mushroom Phellinus liteus.. Chem Pharm Bull (Tokyo).

[2] Han SB, Lee CW, Jeon YJ, Hong ND, Yoo ID (1999). The inhibitory effect of polysaccharide isolated from Phellinus linteus on tumor growth and metastasis.. Immunopharmacology.

[3] Borchers AT, Stern JS, Hackman RM, Keen CL, Gershwin ME (1999). Mushrooms, tumors and immunity.. Proc Soc Exp Biol Med.

[4] Chihara G, Maeda Y, Hamuro J, Sasaki T, Fukuoka F (1969). Inhibition of mouse sarcoma 180 by polysaccharides from Lentinus edodes (Berk.) sing.. Nature.

[5] Wasser SP (2002). Medicinal mushrooms as a source of antitumor and immunomodulating polysaccharides.. Appl Microbiol Biotechnol.

[6] Guo J, Zhu T, Collins L, Xiao ZX, Kim SH (2007). Modulation of lung cancer growth arrest and apoptosis by Phellinus Linteus.. Mol Carcinog.

[7] Collins L, Zhu T, Guo J, Xiao ZJ, Chen CY (2006). Phellinus liteus sensitizes apoptosis induced by doxorubicin in prostate cancer.. Br J Cancer.

[8] Schmitz I, Kirchhoff S, Krammer PH (2000). Regulation of death receptor-mediated apoptosis pathways.. Int J Biochem Cell Biol.

[9] Shi Y (2002). Mechanisms of caspase activation and inhibition during apoptosis.. Mol Cell.

[10] Thornberry NA, Rano TA, Peterson EP, Rasper DM, Timkey T (1997). A combinatorial approach defines specificities of members of the caspase family and granzyme B. Functional relationships established for key mediators of apoptosis.. J Biol Chem.

[11] Thornberry NA, Lazebnik Y (1998). Caspases: enemies within.. Science.

[12] Susin SA, Lorenzo HK, Zamzami N, Marzo I, Snow BE (1999). Molecular characterization of mitochondrial apoptosis-inducing factor.. Nature.

[13] Zhu T, Guo J, Collins L, Kelly J, Xiao ZJ (2007). Phellinus linteus activates different pathways to induce apoptosis in prostate cancer cells.. Br J Cancer.

[14] Tomayko MM, Reynolds CP (1989). Determination of subcutaneous tumor size in athymic (nude) mice.. Cancer Chemother Pharnacol.

[15] Herr I, Debatin, KM (2001). Cellular stress response and apoptosis in cancer therapy.. Blood.

[16] Breckenridge DG, Germain M, Mathai JP, Nguyen M, Shore GC (2003). Regulation of apoptosis by endoplasmic reticulum pathways.. Oncogene.

